# Homozygous haplotype deficiency reveals deleterious mutations compromising reproductive and rearing success in cattle

**DOI:** 10.1186/s12864-015-1483-7

**Published:** 2015-04-18

**Authors:** Hubert Pausch, Hermann Schwarzenbacher, Johann Burgstaller, Krzysztof Flisikowski, Christine Wurmser, Sandra Jansen, Simone Jung, Angelika Schnieke, Thomas Wittek, Ruedi Fries

**Affiliations:** Lehrstuhl fuer Tierzucht, Technische Universitaet Muenchen, 85354 Freising, Germany; ZuchtData EDV-Dienstleistungen GmbH, 1200 Vienna, Austria; Clinic for Ruminants, University of Veterinary Medicine Vienna, 1210 Vienna, Austria; Lehrstuhl fuer Biotechnologie der Nutztiere, Technische Universitaet Muenchen, 85354 Freising, Germany

**Keywords:** SLC2A2, SUGT1, Homozygous haplotype deficiency, Fanconi-Bickel syndrome, Embryonic lethality

## Abstract

**Background:**

Cattle breeding populations are susceptible to the propagation of recessive diseases. Individual sires generate tens of thousands of progeny *via* artificial insemination. The frequency of deleterious alleles carried by such sires may increase considerably within few generations. Deleterious alleles manifest themselves often by missing homozygosity resulting from embryonic/fetal, perinatal or juvenile lethality of homozygotes.

**Results:**

A scan for homozygous haplotype deficiency in 25,544 Fleckvieh cattle uncovered four haplotypes affecting reproductive and rearing success. Exploiting whole-genome resequencing data from 263 animals facilitated to pinpoint putatively causal mutations in two of these haplotypes. A mutation causing an evolutionarily unlikely substitution in *SUGT1* was perfectly associated with a haplotype compromising insemination success. The mutation was not found in homozygous state in 10,363 animals (P = 1.79 × 10^−5^) and is thus likely to cause lethality of homozygous embryos. A frameshift mutation in *SLC2A2* encoding glucose transporter 2 (GLUT2) compromises calf survival. The mutation leads to premature termination of translation and activates cryptic splice sites resulting in multiple exon variants also with premature translation termination. The affected calves exhibit stunted growth, resembling the phenotypic appearance of Fanconi-Bickel syndrome in humans (OMIM 227810), which is also caused by mutations in *SLC2A2*.

**Conclusions:**

Exploiting comprehensive genotype and sequence data enabled us to reveal two deleterious alleles in *SLC2A2* and *SUGT1* that compromise pre- and postnatal survival in homozygous state. Our results provide the basis for genome-assisted approaches to avoiding inadvertent carrier matings and to improving reproductive and rearing success in Fleckvieh cattle.

**Electronic supplementary material:**

The online version of this article (doi:10.1186/s12864-015-1483-7) contains supplementary material, which is available to authorized users.

## Background

A large proportion of the genes of current cattle breeds can be traced back to a small number of founder animals (*i.e.*, key ancestors), mostly influential breeding bulls that have been widely used in artificial insemination [1,2]. As a result, individuals within current cattle populations are closely related. Intense artificial selection adds to the declining effective population size (N_e_) [3]. In the Fleckvieh population, the estimated N_e_ is 160 [4] and thus somewhat higher compared to the estimates for other dairy breeds [5]. Such structures make cattle populations susceptible to the propagation of recessive disorders. The frequency of recessive deleterious alleles may unnoticeably increase and eventually result in frequent homozygotes with a fatal phenotype.

After the implementation of genome-assisted selection in many cattle breeds [6], at least the male breeding animals are routinely genotyped using dense SNP arrays in order to estimate their genetic value. Comprehensive genotype data also facilitate to unravel the genetic architecture of complex traits (*e.g.*, [7,8]) and to rapidly pinpoint genomic regions underlying mendelian traits [9]. Associated genome regions can be identified in studies involving phenotypically affected and unaffected animals, usually by performing genome-wide association studies followed by autozygosity mapping (*e.g.*, [10]).

However, large-scale genotype data now also allow to identify recessively inherited diseases without observing affected individuals based on the identification of haplotypes with a deficit in homozygous animals [11]. Regions with homozygous haplotype deficiency (HHD) are likely to harbor deleterious mutations, which may compromise pre-, peri- and postnatal survival in homozygous state. HHD has been identified in several cattle breeds, and causal mutations resulting in early embryonic losses have been postulated [2,12-15].

Here we report four regions with HHD in Fleckvieh cattle. Whole-genome sequencing data of 263 individuals from five different breeds facilitated to identify putatively causal mutations for two harmful haplotypes*.* We show that HHD results from early embryonic losses and juvenile mortality, respectively.

## Results

### Homozygous haplotype deficiency in Fleckvieh cattle

Analyzing haplotypes of 25,544 Fleckvieh animals in a sliding window-based scan revealed four regions with HHD (Table 1). These regions were denominated FH1-FH4, with FH being an abbreviation for ‘*Fleckvieh haplotype*’. Homozygous animals were absent for FH1 and FH4, although 20 and 33, respectively, were expected (P = 4.81 × 10^−9^ and P = 1.26 × 10^−14^). Insemination success was reduced by 6.64% and 5.99% in risk-matings (sire and dam sire are carriers) of FH1 and FH4, respectively. This decline is close to the expectation of 6.25%, when assuming a recessive embryonically lethal mutation and an average insemination success of 50% in Fleckvieh cattle (0.5 × 0.125 = 0.0625).

**Table 1:** **Four regions with homozygous haplotype deficiency**

**Abbreviation**	**Chr**	**Start position**	**End position**	**Fq.**	**Homozygous animals**			**Number of matings**		**Difference in insemination success**		**Difference in stillbirth rate**		**Difference in juvenile mortality**		
					**exp**	**obs**	**P**	**non-risk**	**risk**	**%**	**P**	**%**	**P**	**Day 10**	**Day 365**	**P**
FH1	1	1,668,494	6,187,555	2.9	20	0	4.8 × 10^−9^	164,324	14,013	−6.64	7.1 × 10^−25^	−0.4	0.64	−0.3	0.4	0.004
FH2	1	96,169,900	97,123,740	4.1	37	2	1.2 × 10^−13^	109,386	8,428	−1.57	0.014	0.7	0.53	1.6	6.6	2.9 × 10^−41^
FH3	10	26,929,817	35,479,280	3.3	41	3	2.1 × 10^−14^	136,286	10,936	−4.06	2.4 × 10^−17^	1.4	0.12	1.8	4.3	1.3 × 10^−26^
FH4	12	10,859,759	12,805,107	3.3	33	0	1.3 × 10^−14^	86,464	4,793	−5.99	2.9 × 10^−11^	0.4	0.39	0.5	0.7	0.34

Insemination success, the proportion of stillborn calves and juvenile mortality of descendants from risk matings were analyzed for four haplotypes with a significant deficit in homozygous animals. The physical position of the deleterious haplotypes was obtained based on the UMD3.1 assembly of the bovine genome.

**Figure 1:**
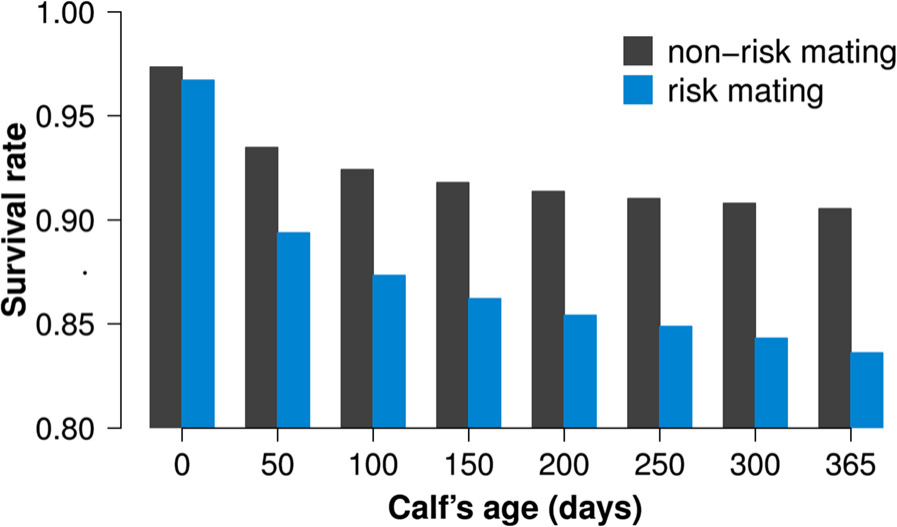
Survival rate of descendants from FH2 risk matings. Bars represent the first-year survival rate of 8,428 and 109,386 descendants from FH2 risk (blue) and non-risk (darkgrey) matings, respectively.

Two and three animals homozygous for FH2 and FH3, respectively, were observed, although 37 and 41 were expected (P = 1.16 × 10^−13^ and P = 2.14 × 10^−14^). A decline in insemination success (−4.06%, P = 2.4 × 10^−17^) and a reduced first-year survival rate of progeny (−4.3%, P = 1.3 × 10^−26^) was observed in FH3 risk matings, indicating both increased pre- and postnatal mortality. Insemination success and stillbirth rate were not affected in FH2 risk matings ruling out HHD to result from increased pre- and perinatal mortality. However, the first-year survival rate of descendants from FH2 risk matings was reduced by 6.6% (P = 2.9 × 10^−41^) compared to the survival rate of progeny from non-risk matings, indicating increased juvenile mortality (Figure 1).

### Genome-wide re-sequencing data reveal two candidate causal mutations for FH2

Re-sequencing data were available for 263 animals from ten different cattle breeds with average sequence coverage of 10.56 fold (ranging from 2.26 to 26.35, Additional file 1). To identify possible causal variants for the increased juvenile mortality of FH2-homozygous animals, we considered 25,176 SNP and 2,431 Indels located within a 2.95 Mb interval on chromosome 1 (95,169,000 bp – 98,123,000 bp). Haplotype analysis revealed eight (out of 145) sequenced Fleckvieh animals to carry FH2, implying a frequency of the deleterious mutation of 2.76% in the sequence-derived Fleckvieh genotypes (8 out of 290 alleles). The average sequence coverage of eight FH2-carriers in the FH2 region was 8.57 fold. Variant calling yielded 15,242 variants that were not homozygous for the alternate allele in 145 sequenced Fleckvieh animals. To account for possible errors in the sequence-derived genotypes (*e.g.,* under-calling of heterozygous genotypes [16]) due to low sequence coverage and imperfect LD between the causative mutation and FH2, respectively, we retained only variants that were heterozygous in at least five (out of eight) sequenced FH2-carriers. This filtering revealed 261 candidate causal variants - only two of them were located in coding regions. These variants are a missense mutation (rs384285149, p.P19S, Chr1:97,309,054 bp) in a highly conserved domain of *EIF5A2* encoding the eukaryotic translation initiation factor 5A-2, and a frameshift mutation (rs379675307) in exon 7 of *SLC2A2*, encoding glucose transporter 2 (GLUT2). Both variants are heterozygous in eight FH2-carriers and homozygous for the reference allele in all other sequenced Fleckvieh animals. Moreover, both variants are homozygous for the reference allele in 1007 non-Fleckvieh animals that have been sequenced for the 1000 bull genomes project [2].

The p.P19S (rs384285149) variant in *EIF5A2* is predicted to be highly damaging to protein function (*SIFT*-score: 0.00; *Polyphen*-score: 0.952; Additional file 2). Differential expression of *EIF5A2* is associated with severe growth retardation and a reduced lifespan in mice [17]. p.P19S is therefore a plausible candidate mutation for an increased juvenile mortality. The *SLC2A2* frameshift variant (rs379675307) was confirmed in eight presumed carriers using Sanger sequencing. Eight nucleotides from the third position of exon 7 are replaced by four nucleotides yielding a net loss of four nucleotides (c.771_778delTTGAAAAGinsCATC). The mutation is expected to alter the reading frame and to change the amino acid sequence from position 258 onwards, resulting in a premature translation termination at position 273 (p.L258fs16). The mutated protein should be shortened by 250 amino acids (48%) and should lack essential domains for glucose affinity and transport [18]. Considering the predicted effects of the mutation and the finding that mutations in *SLC2A2* cause a rare recessive disorder with severe growth retardation in humans (Fanconi-Bickel syndrome, FBS, MIM #227810) [19], the frameshift mutation in bovine *SLC2A2* is also a very plausible candidate for an increased juvenile mortality.

Of 3,305 genotyped healthy adult Fleckvieh bulls, 324 were heterozygous for both, rs384285149 in *EIF5A2* and rs379675307 in *SLC2A2*, with complete LD of the variant sites. Consistent with a recessive disease phenotype, none of the healthy animals is homozygous for the mutant alleles (P = 0.003).

### FH2-homozygous animals are retarded in growth and show liver and kidney defects

**Figure 2:**
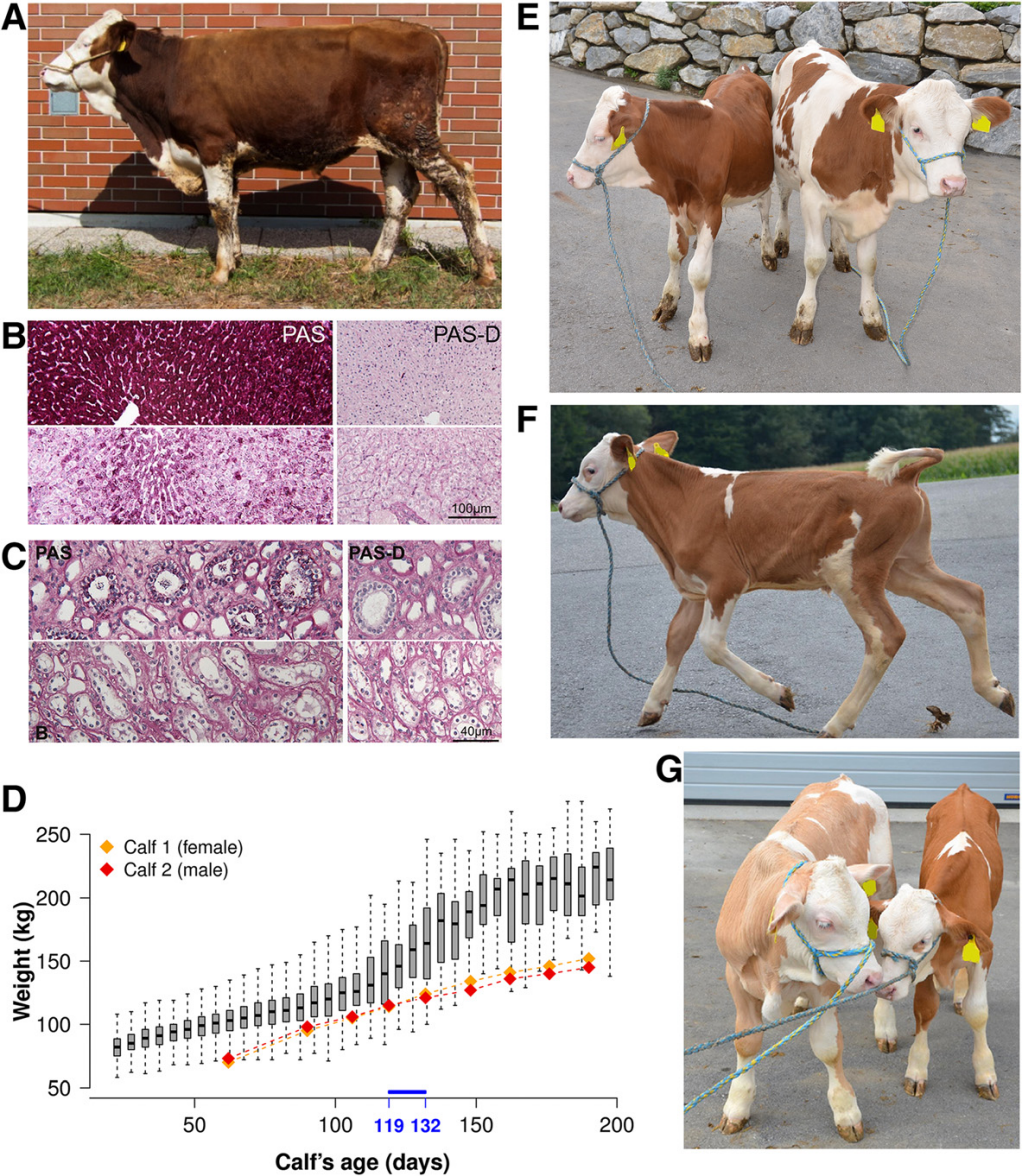
Phenotypic manifestation of the FH2-associated mutation(s). Photograph of a 15-months old Fleckvieh bull being homozygous for the rs379675307 mutation in *SLC2A2* **(A)**. Histological sections of the liver **(B)**. Micrographs of PAS-stained liver specimens from the homozygous animal (upper panel) and from an unaffected control animal (lower panel). PAS-D shows the positive diastase test. Histological sections of the kidney **(C)**. Micrograph of PAS stained kidney specimens from the homozygous animal (upper panel) and from an unaffected control animal (lower panel). PAS-D shows the positive diastase test. Two nineteen weeks old Fleckvieh calves **(E)**. The calf on the left side is homozygous for the *SLC2A2* mutation and the calf on the right side is a healthy animal. Photograph of a nine weeks old Fleckvieh calf homozygous for the *SLC2A2*-mutation **(F)**. Please note the lean body of the FH2-homozygous calf. The affected animal (right side) is compared with a seven weeks (left side) old coeval **(G)**. Figures were kindly supplied by Martina Wassertheurer (NOE Genetik, Austria). The weight of two FH2-homozygous twin calves (orange and red diamonds) is compared to the weight of 74,422 Fleckvieh calves with unknown genotypes (grey boxes) **(D)**. The solid blue line represents the weaning period of the two homozygous calves.

Two male animals were detected to be homozygous for FH2 in the initial scan for HHD. One 15-month old bull was still alive. Sanger sequencing of the bull’s DNA confirmed homozygosity for the rs384285149 variant in *EIF5A2* and for the rs379675307 variant in *SLC2A2*. The growth of the bull was severely retarded although feed intake was reported to be normal (Figure 2A). At the time of admission to the clinic, the bull’s weight was 268.5 kg, which is half of the weight expected at this age for the Fleckvieh breed. Clinical examination did not reveal infectious and chronic diseases. Body temperature, pulse and respiratory rates were within normal ranges. During the hospitalization period of 50 days, the average weight gain was 330 g/day only, although feed intake and rumination were normal. There were no obvious abnormalities of the digestive system (*i.e.*, oral cavity, pharynx, oesophagus, forestomachs).

Analysis of urine samples revealed glucosuria and proteinuria as well as an excessive excretion of electrolytes (potassium, sodium, chloride). Decreased urine specific gravity indicated renal insufficiency (Additional file 3). The measurements of the 12 h water intake (36 l) and urine excretion (34 l) were approximately twice the physiological values.

Analysis of blood parameters revealed an increased glucose level (4.2 mmol/l) but normal insulin concentration (71.8 pmol/l). The activities of liver function related enzymes (aspartate transaminase, glutamate dehydrogenase, gamma-glutamyltransferase) were increased indicating liver cell damage (Additional file 4). Histological sections of liver and kidney biopsies revealed hepatorenal glycogen accumulation (Figure 2B-C). Necropsy findings confirmed liver and kidney defects.

Shortly after the identification of the first two animals homozygous for the *SLC2A2* frameshift mutation, we diagnosed an additional 18 Fleckvieh animals with severe growth retardation that were all homozygous for the *SLC2A2* mutation (and the closely linked *EIF5A2* variant, Figure 2E-G). Two of them, male and female twin calves, were admitted to our research station at the age of 62 days. At 70 kg (female) and 73 kg (male) they were underweight, possibly due to twin pregnancy. However, with an average weight gain of ~760 g/day they developed normal until weaning at 132 days (Figure 2D). After weaning, the weight gain was only ~450 g/day, *i.e*. half of the weight gain of healthy animals of the same age. Apart from the retarded growth, they didn’t show any other obvious clinical abnormalities.

A nine month old Fleckvieh bull with symptoms resembling the condition of the animals examined in the present study was reported in 1996 [20,21]. Inspection of the bull’s pedigree revealed that its sire had been a carrier of FH2. We extracted DNA from a formalin-fixed and paraffin-embedded liver sample provided by the authors. Sanger sequencing confirmed homozygosity for the p.P19S variant in *EIF5A2* and for the p.L258fs16 variant in *SLC2A2.*

### The FH2-associated frameshift mutation in SLC2A2 activates cryptic splice sites

**Figure 3:**
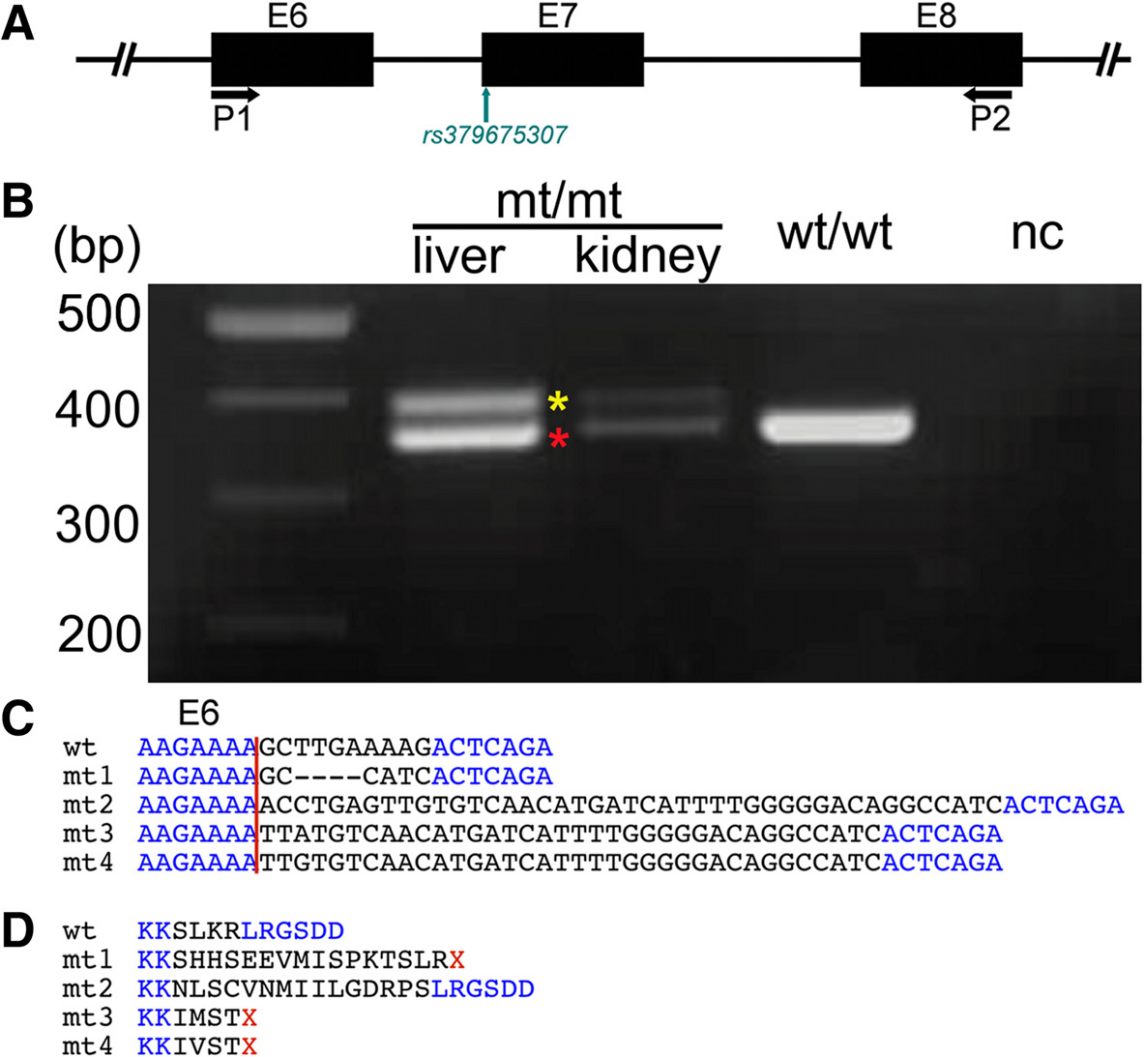
A frameshift mutation in *SLC2A2* induces cryptic splice sites. Schematic representation of exon-intron boundaries of bovine *SLC2A2* **(A)**. RT-PCR primers (P1, P2) were designed to hybridize to exons 6 and 8 (E6, E8). The green arrow indicates the position of rs379675307 at the beginning of exon 7 (E7). Examination of *SLC2A2* mRNA with RT-PCR **(B)**. A 370 bp product was amplified in a liver sample of a homozygous wild type (wt/wt) animal. In liver and kidney samples of the mutant homozygous animal (mt/mt), two RT-PCR products of 370 bp (red asterisk) and 400 bp (yellow asterisk) were observed. The 370 bp RT-PCR fragment corresponds to the c.771_778delTTGAAAAGinsCATC (mt1) variant **(C)**. The 400 bp RT-PCR fragment represents three aberrant nucleotide sequences at the 5′-end of exon 7 with 412 bp (mt2) and 405 bp (mt3, mt4). The red vertical line delineates the last nucleotide of exon 6, blue letters indicate concordant nucleotides and underscores the c.771_778delTTGAAAAGinsCATC variant in four aberrant sequences. Wild type (wt) and mutant (mt1 – mt4) sequence of bovine GLUT2 **(D)**. Blue letters indicate concordant amino acids, red color highlights the premature termination codons in mt1, mt3 and mt4.

The effect of the c.771_778delTTGAAAAGinsCATC variant (rs379675307) on *SLC2A2* transcription was examined by RT-PCR with RNA extracted from liver and kidney biopsies of the homozygous animal (described above) and a wild-type animal. Using primers in exons 6 and 8, we obtained a unique 370 bp RT-PCR product from the homozygous wild-type animal and two RT-PCR products of ~370 bp and ~400 bp from the homozygous mutant animal (Figure 3A-B). The sequence of the wild-type RT-PCR product corresponded to the reference sequence of the bovine *SLC2A2* mRNA and the sequence of the mutant ~370 bp RT-PCR fragment matched the c.771_778delTTGAAAAGinsCATC transcript variant (mt1). In order to obtain the sequence of the ~400 bp fragment, it was necessary to subclone it. Sequence analysis of the subclones revealed three aberrant sequences at the 5′-end of exon 7 with fragment sizes of 412 bp (mt2), 405 bp (mt3), and 405 bp (mt4). Each of these sequences contained the CATC insert that is specific for the mutated variant (Figure 3C). mt2 is predicted to encode an insertion of 15 amino-acids into the second alpha-helix domain of the cytoplasmic loop in GLUT2 (Additional file 5), and mt3 and mt4 to induce a frameshift that severely truncates the resulting protein (Figure 3D). Bioinformatic analysis ([22,23]) revealed that the c.771_778delTTGAAAAGinsCATC mutation abolishes the consensus nucleotide binding sequences for the serine/arginine-rich splicing factors proteins leading to aberrant splicing sites at exon 7 of *SLCA2A* (Additional file 6).

### FH4 carries a missense mutation in *SUGT1* that is likely to cause early embryonic loss

To identify possible causal variants for FH4, we considered 43,676 SNP and 4,087 Indels located within a 3.96 Mb interval on chromosome 12 (9,859,000 bp – 13,805,000 bp). Eleven sequenced Fleckvieh animals are heterozygous carriers of FH4. Their average sequence coverage in the FH4 region was 7.39 fold. To account for possible errors in the sequence-derived genotypes due to low sequence coverage (*e.g.*, under-calling of heterozygous genotypes) and imperfect LD between the causative mutation and FH4, we filtered for variants that were heterozygous in at least eight (out of eleven) sequenced FH4 carriers. This approach revealed 439 candidate causal variants. Among these, 438 variants are located in intronic and intergenic regions; only one variant is positioned in the coding region (rs110793536, Chr12:11,131,497 bp) and in perfect LD with FH4. All 118 sequenced non-Fleckvieh animals are homozygous for the reference allele. Twelve (out of 1149) sequenced animals from Run4 of the 1000 bull genomes project are heterozygous carriers of the alternate allele (eleven Fleckvieh and one Simmental).

**Figure 4:**
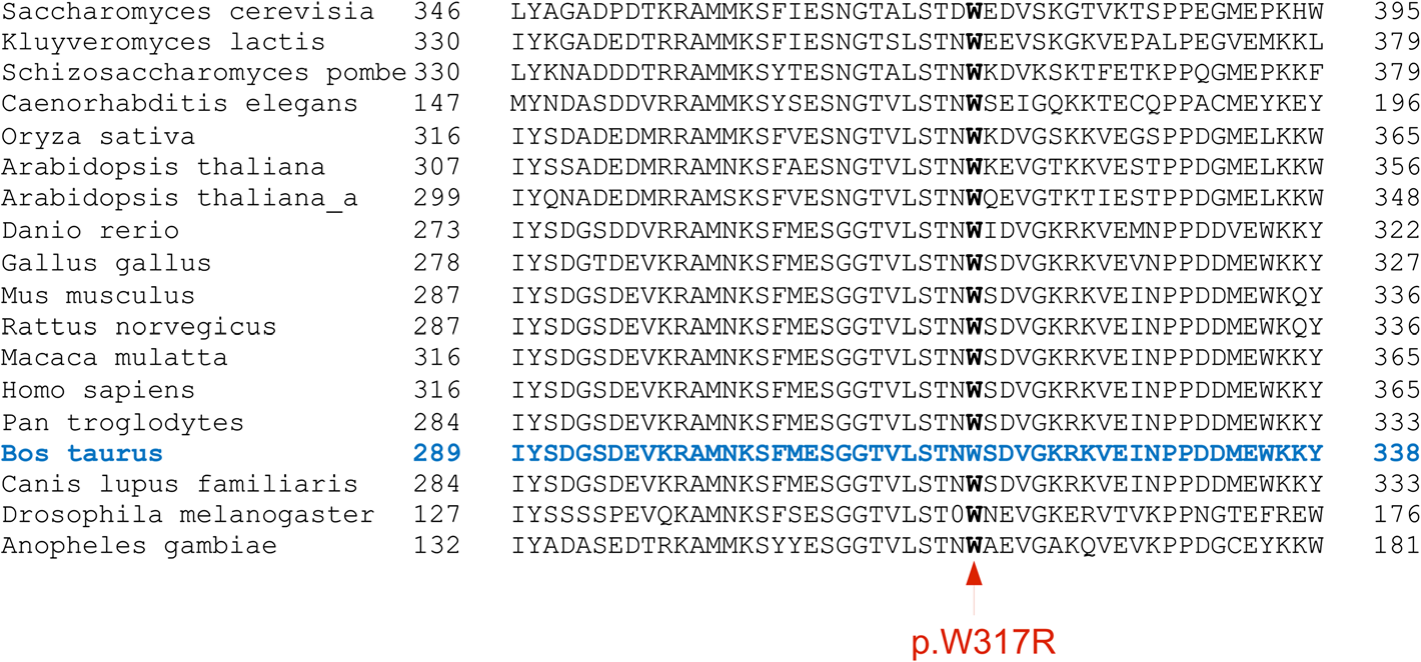
Multi-species alignment of the SGT1 protein sequence. The red arrow indicates p.W317R. Protein sequences were obtained from NCBI for *Saccharomyces cerevisia* (NP_014700.1), *Kluyveromyces lactis* (XP_455814.1), *Schizosaccharomyces pombe* (NP_595340.1), *Caenorhabditis elegans* (NP_505751.1), *Oryza sativa* (NP_001043620.1), *Arabidopsis thaliana* (NP_192865.1, NP_194088.1), *Danio rerio* (NP_001007362.1), *Gallus gallus* (NP_001025994.2), *Mus musculus* (NP_080750.1), *Rattus norvegicus* (NP_001013069.1), *Macaca mulatta* (XP_001084394.1), *Homo sapiens* (NP_001124384.1), *Pan troglodytes* (XP_509801.2), *Bos taurus* (NP_001039668.1), *Canis lupus familiaris* (XP_851986.2), *Drosophila melanogaster* (NP_649783.2) and fc*Anopheles gambiae* (XP_316219.4).

The coding variant (rs110793536) is a missense mutation (p.W317R) in *SUGT1* encoding SGT1, suppressor of G2 allele of SKP1. SGT1 controls kinetochore function during the metaphase of the mitotic cell cycle [24]. Mutations in *SUGT1* inhibit the G1/S and G2/M cell cycle transitions [25] and may thus cause early embryonic lethality in eukaryotes [24]. The p.W317R-variant is predicted to be highly damaging to SGT1 protein function (*SIFT-*score: 0.00; *Polyphen*-score: 1.00). A tryptophan residue at position 317 of SGT1 is conserved throughout the eukaryotes suggesting that it is essential for normal SGT1 protein function (Figure 4).

We obtained genotypes at rs110793536 for 3,411 adult Fleckvieh bulls. Among these, 451 were heterozygous, yielding a minor allele frequency of 6.49%. Consistent with the assumption of embryonic lethality of homozygotes, animals homozygous for the mutant allele were absent in the genotyped sample (P = 3.56 × 10^−5^). We imputed rs110793536 into a larger cohort of 10,363 adult Fleckvieh animals genotyped with 54 K and 700 K SNP arrays using 145 sequenced Fleckvieh animals as reference population [26]. After imputation, 838 animals were identified as heterozygous and none as homozygous, although 17 were expected assuming Hardy-Weinberg equilibrium (P = 1.79 × 10^−5^).

Analysis of inseminations after unsuccessful first inseminations indicated very early pregnancy failure in risk matings. While in risk matings 50.93% of cows have a consecutive insemination within 5 and 32 days after the first insemination, the proportion in non-risk matings is 48.35% (P = 2.9 × 10^−11^).

### Attempt to identifying a causal variant for FH3

Our scan for HHD revealed that the lack of FH3-homozygous adult animals results from embryonic losses and an increased juvenile mortality. Three (out of 25,544) animals of the initial scan were homozygous for FH3. However, these animals were not available for phenotyping as they were already slaughtered and died from an unknown cause at the age of 368 and 395 days, respectively. Analysis of a larger number of genotyped animals revealed an additional five FH3-homozygous calves. Three of them died from an unknown cause within 70 days after birth (Additional file 7). Two live homozygous young bulls were inspected at 209 and 277 days of age. Both animals were apparently healthy, although one of them seemed mildly underdeveloped (Additional file 8). The young bull was admitted to the clinic for in-depth examination. Its weight gain during a ~180 days hospitalization period was normal. There were no indications for any apparent diseases.

We sequenced the genome of the FH3-homozygous animal to coverage of 8.61 fold. The sequence data of the FH3-homozygous animal were analysed together with the sequence data of 263 unaffected animals, among them eight heterozygous FH3 carriers with average sequence coverage of 8.07 fold in the FH3-associated region. To identify possible causal variants we considered 94,783 SNP and 9,662 Indels located within a 10.55 Mb interval on chromosome 10 (25,929,000 bp – 36,479,000 bp). We screened for variants that were homozygous for the alternate allele in the FH3-homozygous animal and heterozygous or homozygous for the reference allele in all other animals. This approach revealed 442 compatible variants (439 non-coding and 1 coding). The coding variant is a missense mutation in *LOC783918* (rs384792959, Chr10: 26,948,606 bp, p.V327I). Thirty-eight (out of 263) sequenced animals including all eight adult FH3-carriers, are heterozygous for rs384792959. However, this variant also segregates in breeds other than Fleckvieh, and two non-Fleckvieh animals from Run4 of the 1000 bull genomes project are homozygous for the alternate allele.

### Attempt to identifying a causal variant for FH1

The lack of FH1-homozygous adult animals most likely results from embryonic losses. We inspected 64,790 SNP and 6,069 Indels located within the 6.52 Mb FH1-interval on chromosome 1 (668,000 bp – 7,187,000 bp). Assuming strict embryonic mortality, the causal variant should not be homozygous for the alternate allele in 263 adult re-sequenced animals. Of 25,217 variants fulfilling this criterion, 24 variants were heterozygous in at least three (out of six) re-sequenced FH1-carriers (accounting for low sequence coverage and *e.g.*, under-calling of heterozygous genotypes) (Additional file 9). Of these 24 variants, nine were never homozygous for the alternate allele in 1149 animals re-sequenced for the 1000 bull genomes project. Due to their location in non-coding regions of the genome, a functional assessment was not readily possible and therefore not attempted.

## Discussion

Scanning for missing homozygosity in 25,544 animals revealed four harmful haplotypes in the Fleckvieh breed. Their frequencies ranged from 2.9 to 4.1%. Identifying less frequent haplotypes with a deficit in homozygous animals requires larger sample sizes. More than 100,000 individuals would be necessary to uncover HHD with P < 0.001 for haplotypes with frequencies below 1%. Haplotypes with missing homozygosity due to embryonic lethality have previously been reported for the Holstein, Jersey, Braunvieh, Montbéliarde, Normande and Ayrshire populations (*e.g.,* [11,14,27]), and causal mutations have been identified for some of them (*e.g.,* [2,12-15]). These haplotypes may cause considerable economic loss as they manifest themselves primarily in fertilization failures or abortions.

Sequence data of 263 animals enabled us to pinpoint candidate causative mutations for two haplotypes with a homozygosity deficit. FH2 on bovine chromosome 1 affects calf survival. Two Fleckvieh-specific mutations were perfectly associated with FH2. The rs384285149 mutation is a missense mutation affecting a highly conserved domain of *EIF5A2.* The rs379675307 mutation causes a frameshift in *SLC2A2* and also activates cryptic splice sites resulting in multiple aberrant exon variants. Three of them induce premature translation termination. A fourth exon variant encodes an insertion of 15 amino-acids in the cytoplasmic loop of SLC2A2 without inducing premature protein termination but possibly preventing normal protein function [28]. Mutations in human *SLC2A2* cause Fanconi-Bickel syndrome (FBS), a rare recessive glycogen storage disorder which results in severe growth retardation [19]. Clinical symptoms, namely growth retardation, laboratory parameters and necropsy findings of an animal homozygous for the bovine *SLC2A2* mutation are similar to those described for human FBS [19]. The analogy of the clinical findings to the human condition provides strong evidence that the loss-of-function mutation in *SLC2A2* causes the disease in cattle. However, due to the complete linkage disequilibrium of the *SLC2A2* and *EIF5A2* mutations, the *EIF5A2* mutation cannot be ruled out to solely cause the disease or to contribute to its progression.

Unraveling the pathophysiology of the condition most likely resulting from the *SLC2A2* mutation requires a thorough understanding of the function of GLUT2, the protein encoded by *SLC2A2*. Besides facilitating bidirectional fluxes of monosaccharides, GLUT2 may also play a role in glucose homeostasis by sensing blood glucose concentrations, inducing insulin secretion and controlling food intake as shown in mice [28,29]. In FBS patients, GLUT2 deficiency results in high postprandial blood glucose concentration as a result of both reduced glucose uptake in hepatocytes and low insulin secretion, probably due to an impaired blood glucose sensing in pancreatic beta cells [30]. In the fasting state, GLUT2 deficiency impedes glucose release from hepatocytes resulting in high glucose concentrations in hepatic cells and low blood glucose levels, which inhibit glycogen degradation [30]. Insufficient glucose utilization may cause the growth retardation of animals being homozygous for the *SLC2A2* frameshift mutation. However, their development during the suckling period is nearly normal. The high abundance of growth factors including insulin in bovine whole milk [31] may promote growth of affected milk-fed calves and compensate the lack of growth stimuli.

Apart from the retarded growth, particularly after weaning, animals homozygous for the *SLC2A2* mutation appear healthy. However, most of them are culled at a juvenile age due to the low growth performance, explaining the lack of FH2-homozygous adult animals. The condition has not been recognized as a genetic disorder so far, although, at a frequency of 0.041, more than 1500 affected animals are expected to be born annually assuming 1,000,000 births per year in the German Fleckvieh population. Interestingly, the disease is not a new phenomenon. It must have been present in the Fleckvieh population for at least two decades as the analysis of a historical sample revealed. Survey programs for genetic defects in cattle focus on the perinatal period and externally visible traits. They don’t allow for recognizing a genetic disorder without obvious perinatal symptoms, as is the case for the FH2-associated condition. Furthermore, here we provide an example for the detection of a disease that compromises calf survival without direct phenotypic clue. To our knowledge, this is unprecedented in a livestock species.

Using sequence data we were also able to identify a mutation that is most likely responsible for the harmful FH4 haplotype on bovine chromosome 12. FH4 affects insemination success in risk matings. The complete absence of homozygous animals indicates an embryonically recessive lethal mutation. A Fleckvieh-specific, evolutionarily unlikely missense mutation in *SUGT1* is perfectly associated with FH4. The mutation is expected to affect cell-cycle progression [25]. A defect in such a fundamental process of development most likely leads to the very early loss of embryos. It is conceivable that homozygously affected zygotes don’t undergo division at all. Thus, the effect of this mutation may manifest itself rather as delayed conception than embryonic lethality. Pregnancy is not initiated and the cow enters the next estrus cycle without delay. Thus the consequences of the *SUGT1* mutation on cattle fertility are less pronounced than, *e.g.*, of a mutation in *UMPS* that leads to embryonic mortality at ~ day 40 of gestation [32].

We were able to detect very plausible candidate causal mutations for two of four haplotypes with reduced or missing homozygosity. We think there are four main reasons that we have not been able to uncover plausible causal variants for FH1 and FH3. Firstly, our algorithm for searching potential causal variants was restricted to well-annotated protein coding regions. Causal variants might be of regulatory nature and might reside in not translated intronic or intergenic genome regions. Secondly, annotation of the bovine genome is often flawed due to assembly problems or gaps in the reference sequence [33]. Causal variants located in such regions will be entirely missed or not recognized to affect protein coding. Thirdly, disease-associated haplotypes may be in incomplete LD with the causative mutation. FH1 and FH3 are considerably larger than FH2 and FH4 for which we identified plausible candidate causal mutations. Our data did not allow us to more precisely narrow down FH1 and FH3. Animals carrying shorter segments of deleterious haplotypes may thus not be detected. Furthermore, ancestral haplotypes without the mutation might persist in the population [34,35]. Both situations result in the erroneous identification of haplotype carriers. Fourthly, structural rearrangements of the genome might be causal for HHD [13]. However, detecting structural variants is notoriously difficult in low- to medium coverage re-sequencing data and different approaches for variant detection may yield inconsistent results [36]. Furthermore, gaps in the reference genome may lead to many false variant calls [37]. Several animals of the present study were sequenced at relatively low fold sequence coverage (Additional file 1). We therefore decided not to apply structural variant discovery.

FH1 and FH4 compromise insemination success in risk matings, most likely due to early embryonic losses, FH2 affects calf survival and FH3 compromises both, insemination and rearing success. However, some FH3-homozygous animals reach adulthood without obvious health problems. The unequivocal identification of the causal mutations and affected gene(s) will be prerequisite for an optimal management of FH3 as such knowledge will provide clues to the pathophysiology underlying the incomplete penetrance and possibly to a therapy. In any case, our results provide the basis for the highly reliable identification of carriers of all four harmful haplotypes. Since most male breeding animals are routinely genotyped for genetic evaluation, the carrier status is directly available for them. The carrier status of the female mating partners can be derived from their genotyped male ancestors. However, genotyping of females would enable a more accurate identification of carrier animals. The consequent avoidance of carrier matings will contribute to better reproductive success and to a reduction of calf loss.

## Conclusions

Scanning for missing homozygosity enabled us to identify four haplotypes with a significant deficit in homozygous adult animals. Missing or reduced homozygosity results from increased pre- and postnatal mortality of homozygotes. A frameshift mutation in *SLC2A2* is associated with a harmful haplotype that compromises survival of homozygous animals. The mutation impairs glucose utilization of homozygous animals and thereby causes stunted growth. Another missense mutation in *SUGT1* is perfectly associated with a haplotype causing early embryonic losses. Our results provide the basis for genome-assisted mating programs to avoiding inadvertent carrier matings and to improving reproductive and rearing success in Fleckvieh cattle.

## Methods

### Ethics statement

DNA for genotyping and sequencing was prepared from semen samples of artificial insemination (AI) bulls. Semen samples were collected by approved commercial AI stations as part of their regular breeding and reproduction measures in cattle industry. The homozygous animal was examined at the animal clinic of the veterinary university of Vienna as part of their regular practice. All homozygous animals result from carrier matings, that inadvertently happened in Fleckvieh farms. No ethical approval was required for this study.

### Animals, genotypes, quality control and haplotype inference

The animals for the HHD scanning were genotyped with the Illumina BovineSNP50 Bead chip (version 1 and version 2) comprising approximately 54,000 SNP. The chromosomal position of the SNP was determined based on the UMD3.1 assembly of the bovine genome [38]. Only autosomal SNP were considered. Animals and SNPs with call-rate below 95% were excluded, as well as SNP with minor allele frequency below 2% and SNP deviating significantly (P < 10^−6^) from the Hardy-Weinberg equilibrium. The pedigree-based relationship of the animals was compared with the realized genomic relationship [39] and animals with major discrepancies were excluded. Genotypes of sire-offspring pairs were inspected for mendelian errors (*i.e.,* genotype AA and BB in sire and offspring, respectively) and SNP with more than 500 mendelian errors were excluded. The final dataset contained 25,544 animals and 41,251 autosomal SNP with an average per-individual call-rate of 99.61%. Haplotypes were inferred and sporadically missing genotypes were imputed using *BEAGLE* genetic analysis software [40]. Custom R scripts were used to correct phasing errors in haplotypes in large half-sib families (more than ten members).

### Identification of homozygous haplotype deficiency

A sliding window with variable size (ranging from 0.75 to 10 Mb) was shifted along each chromosome (in steps of 0.5 × window size). Within each sliding window, haplotypes with a frequency above 2% were retained for the identification of HHD. The expected number of homozygous animals was calculated using haplotype information from sire, maternal grandsire and haplotype frequency. An exact binomial test was applied to compare the observed number of homozygous animals with its expectation. Four haplotypes with a significant deficit of homozygous animals (P < 1 × 10^−6^) were inspected for harmful phenotypic effects.

### Phenotypic effects associated with harmful haplotypes

Only few females have already been genotyped in the Fleckvieh breeding population. To inspect the impact of HHD on fertility, we scrutinized the insemination success where carrier bulls were mated to cows descending from carrier sires (*i.e.* risk matings). Insemination success was analyzed using records of 4,226,431 artificial inseminations carried out between bulls with known haplotype status and cows descending from sires with known haplotype status. The insemination success in risk matings was compared with the insemination success in non-risk matings (non-carrier bulls mated to daughters of carrier sires) using a linear regression model.

Stillbirth incidence and juvenile mortality were analyzed in 2,335,836 calvings with known haplotype states from sires and maternal grandsires. Records of calves leaving the recording systems within 365 days after birth for other reasons than death (*e.g.,* export to other country) were omitted. A Kaplan-Meier estimator [41] was obtained by contrasting the survival rate of calves descending from risk matings with the survival rate of calves descending from non-risk matings.

### Generation of sequence data

Genomic DNA of 220 animals (102 Fleckvieh, 34 Braunvieh, 50 Holstein, 5 Northern Finncattle, 15 Original Simmental, 12 Gelbvieh, 2 Ayrshire) was prepared from semen samples following standard protocols using proteinase K digestion and phenol-chloroform extraction. Paired-end libraries were prepared using the paired-end TruSeq DNA sample prep kit (Illumina inc., San Diego, CA, USA) and sequenced using the Illumina HiSeq 2500 instrument (Illumina inc., San Diego, CA, USA). The resulting reads (read length 101 bp) were processed with the Illumina BaseCaller during the sequencing step. The alignment of the reads to the University of Maryland reference sequence (UMD3.1) [38] was performed with *BWA* [42]. The resulting per individual SAM files were converted into BAM files with *SAMtools* [43]. Duplicate reads were identified and marked with the MarkDuplicates command of *Picard* (http://picard.sourceforge.net/).

### Variant calling, imputation and annotation

Sequence data of another 43 Fleckvieh animals [1] were additionally exploited for variant calling. Polymorphic sites including short insertions and deletions were identified in all 263 sequenced animals simultaneously using the multi-sample approach implemented in *SAMtools* along with *BCFtools* [43]. *BEAGLE* phasing and imputation was used to improve the primary genotype calling by *SAMtools*. The functional effects of the variants were predicted based on the gene annotation of the UMD3.1 assembly of the bovine genome as described [1]. The consequences of amino acid substitutions on protein function were predicted using *PolyPhen-2* [44] and *SIFT* [45].

### Identification of candidate causal variants

Variants located within each associated haplotype ±1 Mb on either side were considered for the identification of possible causal mutations. Additionally, the sequence-derived genotypes of 1149 animals from Run4 of the 1000 bull genomes project [2] were analyzed to obtain the genotype distribution of associated variants in different breeds.

### Validation of three identified polymorphisms

PCR primers were designed to scrutinize the rs379675307, rs384285149 and rs110793536 polymorphisms by classical Sanger sequencing (Additional file 10). Genomic PCR products were sequenced using the BigDye® Terminator v1.1 Cycle Sequencing Kit (Life Technologies) on the ABI 3130x1 Genetic Analyzer (Life Technologies). Genotypes for *rs379675307*, rs384285149 and rs110793536 were obtained by KASP (LGC Genomics) and TaqMan® (Life Technologies) genotyping assays (Additional file 10).

### Clinical examination of an affected animal

A 15 month old FH2-homozygous Fleckvieh bull with severe growth retardation was admitted to the animal clinic. Initial examination (including weighing) was performed upon admission of the young bull. Urine and blood samples were taken twice during the hospitalization period of 50 days. Laboratory parameters were obtained and compared with bovine reference values [46]. Ultrasound guided samples of the liver and right kidney were taken, fixed in 4% buffered formalin and embedded in paraffin. Sections were stained with Periodic Acid Schiff (PAS) for the detection of glycogen (PAS staining kit # 12153, Morphisto, Germany) and consecutive sections were stained with PAS after diastase digestion (PAS-D) for 1 h at 37°C, an enzyme that breaks down glycogen. Due to the advanced state of the disease and with no prospect for improvement, the young bull was euthanized and subjected to necropsy after fifty days of hospitalization.

### RT-PCR

Total RNA from liver biopsy was extracted using Trizol (Invitrogen) according to the manufacturer’s protocol. After *DNase*I treatment (Fermentas), RNA was quantified using a NanoDrop ND-1000 (NanoDrop Technologies) spectrophotometer, and RNA integrity determined by denaturing EtBr 1% agarose gel electrophoresis. Complementary DNA (cDNA) was synthesized using the First Strand cDNA Synthesis Kit (Fermentas).

*SLC2A2* mRNA was examined by RT-PCR using primers 1F – CCTGGGCAATCACGAGCTAT and 1R - TCCAGCTGTCTGGAAAATGC, which hybridize to exon 6 and exon 8 and amplify a 370 bp product based on the mRNA reference sequence (NM_001103222) of bovine *SLC2A2*. RT-PCR was performed in 20 μl reaction volumes containing diluted first-strand cDNA equivalent to 50 ng input RNA. PCR products were loaded on 2% agarose gels. For Sanger sequencing, RT-PCR fragments were cloned into the pGEMT-easy PCR cloning vector.

### Topology prediction for bovine GLUT2

The genomic structure of bovine *SLC2A2* was predicted based on the UMD3.1 assembly of the bovine genome sequences [38] and the Dana-Farber Cancer Institute bovine gene index release 12.0 [47] by using the *GENOMETHREADER* software tool [48]. The *GENOMETHREADER* output was viewed and edited using the *Apollo* sequence annotation editor [49]. The protein topology of bovine GLUT2 was predicted using the *Protter* software tool [50]. Multi-species alignment of protein sequences was performed using the *ClustalW* tool [51]. The coordinates of extra- and intracellular elements were inferred based on the results of crystal structure modeling of GLUT1-4 homologues [52].

### Availability of supporting data

The sequence data of 43 Fleckvieh animals are publically available in the Sequence read Archive of NCBI (http://www.ncbi.nlm.nih.gov/sra) under accession numbers SRX527690-SRX527732. Sequence data of all other animals are part of the upcoming Run5 of the 1000 bull genomes project (http://www.1000bullgenomes.com).

## Additional files

Additional file 1: Figure S1. Sequence-based genotype quality. The concordance between array-based and sequence-derived genotypes was calculated based on 39,624 SNP on chromosome 1 **(A)**. Array-based genotypes (777K) of 133 sequenced Fleckvieh animals were compared with sequence-derived genotypes before (orange) and after (blue) *BEAGLE* imputation. Average concordance before and after *BEAGLE* imputation was 96.19% and 99.93%, respectively. Allele frequency distribution of 157,844 coding variants **(B)**. The functional significance of non-synonymous variants was predicted with *Polyphen-2*. Principal component analysis in 263 sequenced animals genotyped for 24,727,286 autosomal variants **(C)**. Breed of the sequenced animals: Ayrshire (RDC), Fleckvieh, Holstein (HOL), Red-Holstein (RED), Gelbvieh (GV), original Braunvieh (OBV), Braunvieh (BV), Brown Swiss (BSW), Simmental (SIM) and Nordic Finncattle (NFC). Additional file 2: Figure S2. Multi-species alignment of the EIF-5A2 protein sequence. Protein sequence of the eukaryotic translation initiation factor 5A-2. Red color indicates the p.P19S-variant (rs384285149). Protein sequences were obtained from NCBI for *Homo sapiens* (NP_065123.1), *Mus musculus* (NP_808254.1), *Rattus norvegicus* (NP_001094167.1), *Gallus gallus* (NP_990863.1), *Danio rerio* (NP_998427.1), *Callithrix jacchus* (JAB34670.1), *Macaca mulatta* (NP_001247868.1), *Pongo abelii* (NP_001127495.1), *Pan troglodytes* (XP_001163733.1), *Canis lupus familiares* (XP_862075.1), *Drosophila melanogaster* (NP_726411.1), *Anopheles gambiae* (XP_564212.2), *Caenorhabditis elegans* (NP_495807.1) and *Bos taurus* (NP_001179018.1). Additional file 3: Table S1. Analysis of urine samples of the FH2-homozygous animal. Additional file 4: Table S2. Analysis of blood parameters of the FH2-homozygous animal. Bold type indicates anomalous values. Additional file 5: Figure S3. Topology of bovine GLUT2. Bovine GLUT2 consists of 522 amino acids organized in twelve transmembrane domains (TM), a large extracellular loop connecting TM1 with TM2 and a large cytoplasmic loop connecting TM6 with TM7 **(A)**. Orange and blue symbols represent extra- and intracellular alpha helices. The *SLC2A2* frameshift mutation activates cryptic splice sites resulting in four aberrant GLUT2 variants **(B-E)**. Among those, mt1, mt3 and mt4 severely truncate (48%) the resulting protein, whereas mt2 encodes a 15-amino acid insert (green) into the second intracellular alpha helix. Additional file 6: Figure S4. Prediction of exonic splicing enhancers. Exonic splicing enhancers were predicted for the wildtype and for the c.771_778delTTGAAAAGinsCATC sequence using *RESCUE-ESE* **(A, B)** and *ESEfinder3.0* **(C, D)**. Additional file 7: Table S3. FH3-homozygous animals. Three FH3-homozygous animals of our initial HHD scan were already slaughtered and perished for unknown reasons, respectively (grey background). Five additional FH3-homozygous animals were identified exploiting a larger dataset. Three of them perished for unknown reasons. Two animals were inspected at the age of 277 (animal 4) and 209 (animal 7) days. Animal 4 was admitted to the clinic for in-depth examination. Additional file 8: Figure S5. Nine months old FH3-homozygous young bull. The young bull was inspected at nine months of age. According to the possessing farmer, the young bull never suffered from any obvious diseases. Feed intake was reported to be normal. Based on the chest circumference (140 cm), the weight of the young bull was estimated to 230 kg, which is 70 kg lower compared to the weight expected at that age. Additional file 9: Table S4. Twenty-four variants in LD with FH1. Chromosomal coordinates (based on the UMD3.1-assembly) of 24 variants in LD with FH1. Additional file 10: Table S5. Primer sequences.

## Abbreviations

FBS: Fanconi-Bickel syndrome
FH: Fleckvieh haplotype
HHD: homozygous haplotype deficiency
GLUT2: Glucose transporter 2
LD: Linkage disequilibrium
N_e_: Effective population size
OMIM: Online Mendelian Inheritance in Man
PAS: Periodic Acid Schiff
PAS-D: PAS after diastase digestion
RT-PCR: Reverse transcription polymerase chain reaction
SGT1: Suppressor of G2 allele of SKP1
SKP1: S-phase kinase-associated protein 1
SNP: Single nucleotide polymorphism
