# Supplementary figures and images for: Homozygous haplotype deficiency reveals deleterious mutations compromising reproductive and rearing success in cattle
Source: BMC Genomics. 2015 Apr 18;16(1):312. doi: 10.1186/s12864-015-1483-7 (PMC4403906; doi:10.1186/s12864-015-1483-7)

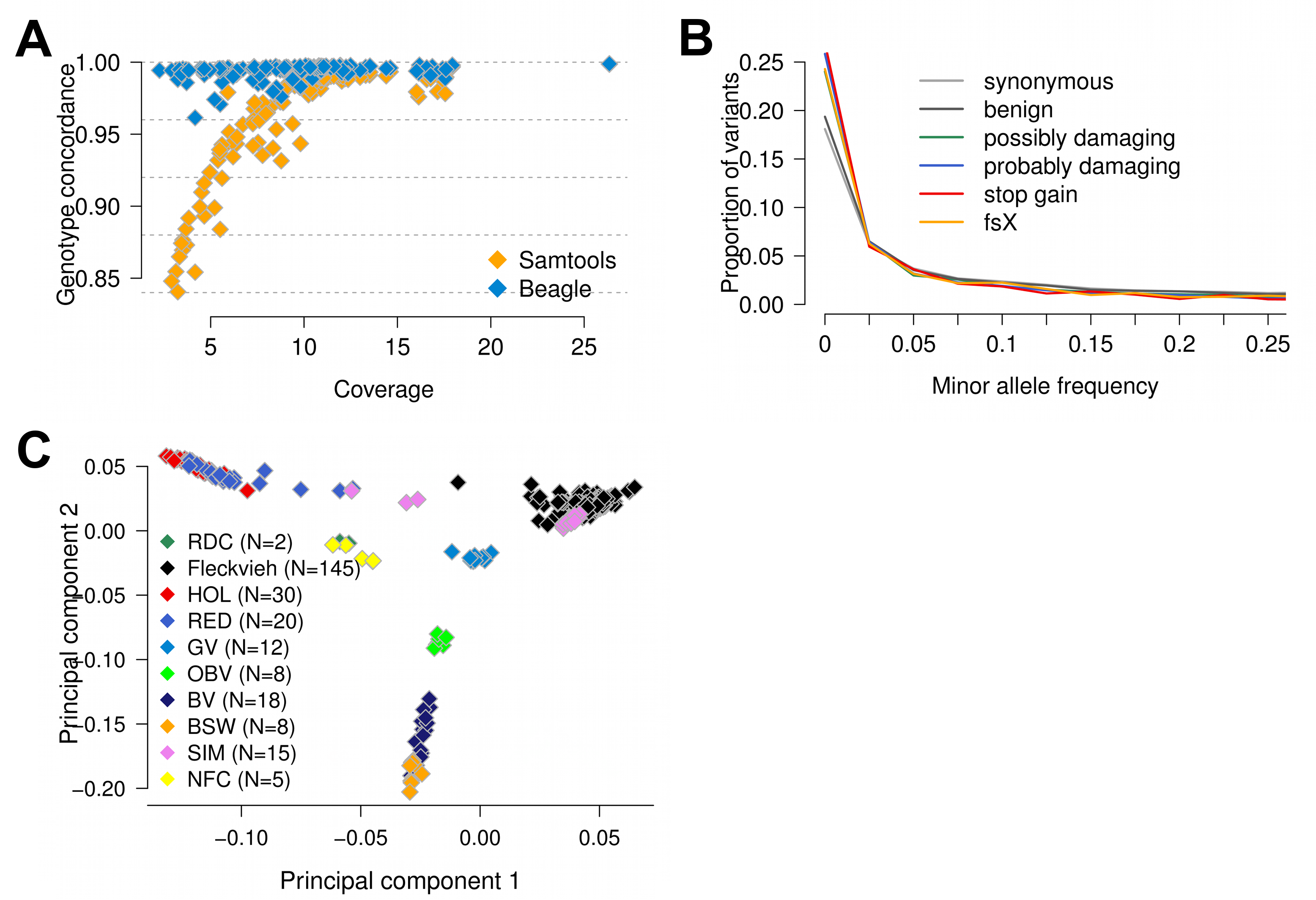

Supplement: Additional file 1: Figure S1. — Sequence-based genotype quality. The concordance between array-based and sequence-derived genotypes was calculated based on 39,624 SNP on chromosome 1 (A). Array-based genotypes (777K) of 133 sequenced Fleckvieh animals were compared with sequence-derived genotypes before (orange) and after (blue) BEAGLE imputation. Average concordance before and after BEAGLE imputation was 96.19% and 99.93%, respectively. Allele frequency distribution of 157,844 coding variants (B). The functional significance of non-synonymous variants was predicted with Polyphen-2. Principal component analysis in 263 sequenced animals genotyped for 24,727,286 autosomal variants (C). Breed of the sequenced animals: Ayrshire (RDC), Fleckvieh, Holstein (HOL), Red-Holstein (RED), Gelbvieh (GV), original Braunvieh (OBV), Braunvieh (BV), Brown Swiss (BSW), Simmental (SIM) and Nordic Finncattle (NFC). [file 12864_2015_1483_MOESM1_ESM.tif]

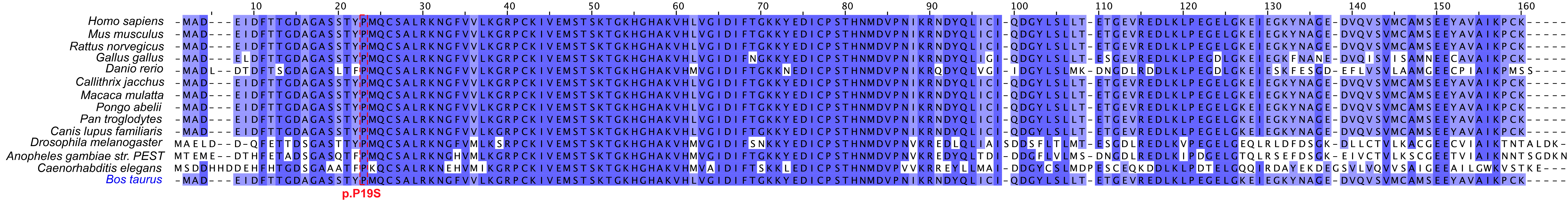

Supplement: Additional file 2: Figure S2. — Multi-species alignment of the EIF-5A2 protein sequence. Protein sequence of the eukaryotic translation initiation factor 5A-2. Red color indicates the p.P19S-variant (rs384285149). Protein sequences were obtained from NCBI for Homo sapiens (NP_065123.1), Mus musculus (NP_808254.1), Rattus norvegicus (NP_001094167.1), Gallus gallus (NP_990863.1), Danio rerio (NP_998427.1), Callithrix jacchus (JAB34670.1), Macaca mulatta (NP_001247868.1), Pongo abelii (NP_001127495.1), Pan troglodytes (XP_001163733.1), Canis lupus familiares (XP_862075.1), Drosophila melanogaster (NP_726411.1), Anopheles gambiae (XP_564212.2), Caenorhabditis elegans (NP_495807.1) and Bos taurus (NP_001179018.1). [file 12864_2015_1483_MOESM2_ESM.tif]

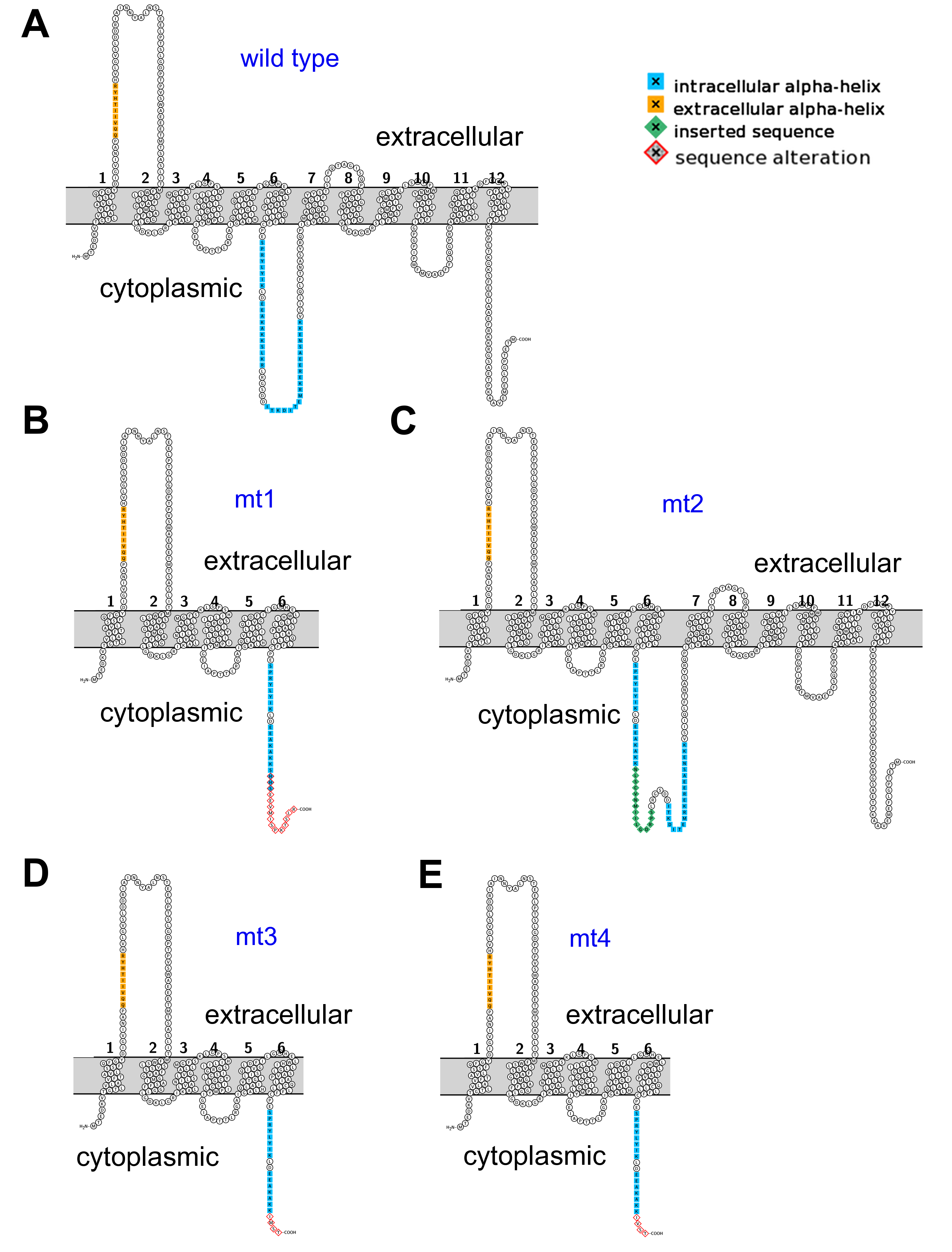

Supplement: Additional file 5: Figure S3. — Topology of bovine GLUT2. Bovine GLUT2 consists of 522 amino acids organized in twelve transmembrane domains (TM), a large extracellular loop connecting TM1 with TM2 and a large cytoplasmic loop connecting TM6 with TM7 (A). Orange and blue symbols represent extra- and intracellular alpha helices. The SLC2A2 frameshift mutation activates cryptic splice sites resulting in four aberrant GLUT2 variants (B-E). Among those, mt1, mt3 and mt4 severely truncate (48%) the resulting protein, whereas mt2 encodes a 15-amino acid insert (green) into the second intracellular alpha helix. [file 12864_2015_1483_MOESM5_ESM.tif]

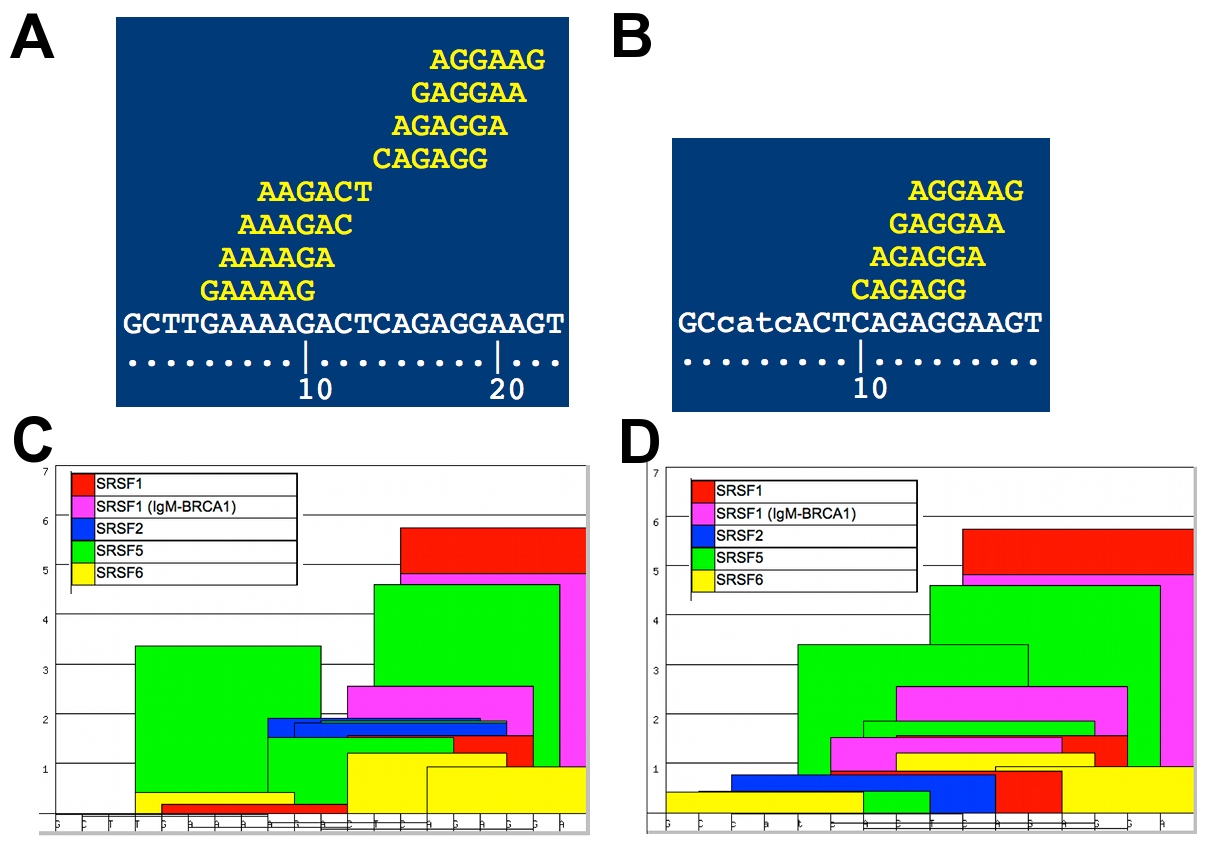

Supplement: Additional file 6: Figure S4. — Prediction of exonic splicing enhancers. Exonic splicing enhancers were predicted for the wildtype and for the c.771_778delTTGAAAAGinsCATC sequence using RESCUE-ESE (A, B) and ESEfinder3.0 (C, D). [file 12864_2015_1483_MOESM6_ESM.tif]

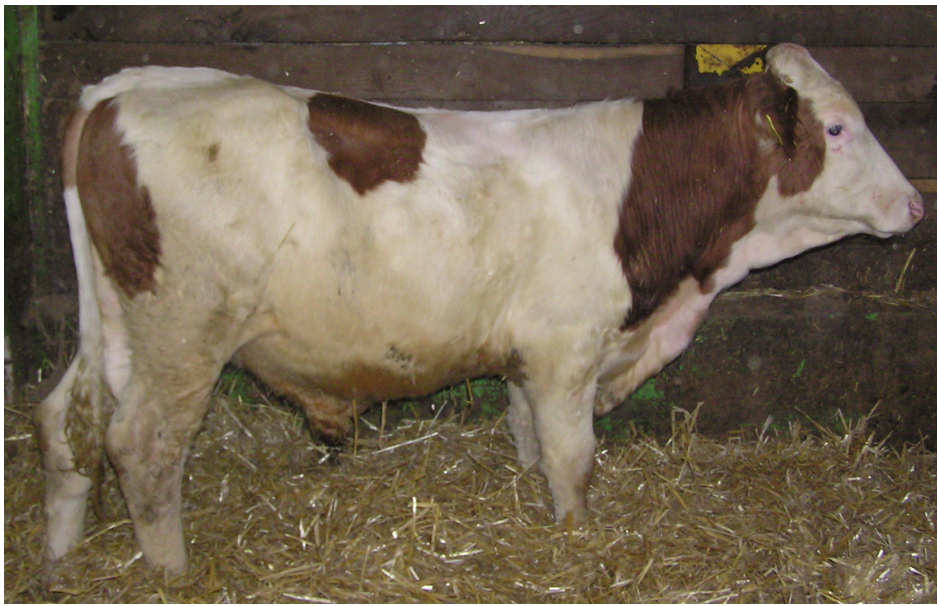

Supplement: Additional file 8: Figure S5. — Nine months old FH3-homozygous young bull. The young bull was inspected at nine months of age. According to the possessing farmer, the young bull never suffered from any obvious diseases. Feed intake was reported to be normal. Based on the chest circumference (140 cm), the weight of the young bull was estimated to 230 kg, which is 70 kg lower compared to the weight expected at that age. [file 12864_2015_1483_MOESM8_ESM.pdf]
