# Supplementary material for: Homozygous haplotype deficiency reveals deleterious mutations compromising reproductive and rearing success in cattle
Source: BMC Genomics. 2015 Apr 18;16(1):312. doi: 10.1186/s12864-015-1483-7 (PMC4403906; doi:10.1186/s12864-015-1483-7)
Supplement: Additional file 3: Table S1. — Analysis of urine samples of the FH2-homozygous animal. [file 12864_2015_1483_MOESM3_ESM.pdf]

Parameter	Unit	Day 1	Day 7	Reference (adult cattle)*
Colour		Light yellow, slightly	Light yellow	Light/dark yellow
Urine specific gravity	g/l	1014	1011	1020 – 1040
pH	value	7	7	7.0 – 8.4
Glucose	mmol/l	65.05	40.35	0
Protein	mg/l	203	161	0
Creatinine	µmol/l	2033.25	1069.66	2200 - 7100

*Kraft W (2005) *Klinische Labordiagnostik in der Tiermedizin* (Schattauer Verlag).
