# Supplementary material for: Homozygous haplotype deficiency reveals deleterious mutations compromising reproductive and rearing success in cattle
Source: BMC Genomics. 2015 Apr 18;16(1):312. doi: 10.1186/s12864-015-1483-7 (PMC4403906; doi:10.1186/s12864-015-1483-7)
Supplement: Additional file 4: Table S2. — Analysis of blood parameters of the FH2-homozygous animal. Bold type indicates anomalous values. [file 12864_2015_1483_MOESM4_ESM.pdf]

Parameter	Unit	Value	Reference (adult cattle) (*)
Glucose	mmol/l	**4.2**	2.2 – 3.3
Creatinine	µmol/l	97.24	88-177
Total protein	g/l	68	60 - 80
Albumin	g/l	35	30-42
AST	µkat/l	**1.36**	<1.34
GLDH	µkat/l	**2.64**	<0.50
GGT	µkat/l	**1.73**	<0.834
Bilirubin (total)	µmol/l	2.22	<5.00
Cholesterol	mmol/l	4.22	>2.00
NEFA	mmol/l	0.19	0.4
Insulin	pmol/l	71.8	57.72 – 101

* Oetzel GR (2004) Monitoring and testing dairy herds for metabolic disease. *Vet Clin North Am Food Anim Pract* 20:651–674.

Blum JW, Kunz P, Bachmann C, Colombo JP (1981) Metabolic effects of fasting in steers. *Res Vet Sci* 31:127–129.

Kraft W (2005) *Klinische Labordiagnostik in der Tiermedizin* (Schattauer Verlag).
