# Supplementary material for: Homozygous haplotype deficiency reveals deleterious mutations compromising reproductive and rearing success in cattle
Source: BMC Genomics. 2015 Apr 18;16(1):312. doi: 10.1186/s12864-015-1483-7 (PMC4403906; doi:10.1186/s12864-015-1483-7)
Supplement: Additional file 7: Table S3. — FH3-homozygous animals. Three FH3-homozygous animals of our initial HHD scan were already slaughtered and perished for unknown reasons, respectively (grey background). Five additional FH3-homozygous animals were identified exploiting a larger dataset. Three of them perished for unknown reasons. Two animals were inspected at the age of 277 (animal 4) and 209 (animal 7) days. Animal 4 was admitted to the clinic for in-depth examination. [file 12864_2015_1483_MOESM7_ESM.pdf]

Animal	Birth date	Day of death	Age of death (days)	Reason of death
Animal 1	27/07/2011	01/10/2012	432	slaughtered
Animal 2	25/03/2012	24/04/2013	395	perished
Animal 3	26/04/2012	29/04/2013	368	perished
Animal 4	28/02/2013	-	-	-
Animal 5	28/02/2013	08/05/2013	69	perished
Animal 6	30/04/2013	10/06/2013	41	perished
Animal 7	07/05/2013	-	-	-
Animal 8	25/08/2013	28/10/2013	64	perished
