# Supplementary material for: Homozygous haplotype deficiency reveals deleterious mutations compromising reproductive and rearing success in cattle
Source: BMC Genomics. 2015 Apr 18;16(1):312. doi: 10.1186/s12864-015-1483-7 (PMC4403906; doi:10.1186/s12864-015-1483-7)
Supplement: Additional file 9: Table S4. — Twenty-four variants in LD with FH1. Chromosomal coordinates (based on the UMD3.1-assembly) of 24 variants in LD with FH1. [file 12864_2015_1483_MOESM9_ESM.pdf]

Chromosom	Position	Gene	Effect	Homozygous in 1000 bull genomes data
1	2344437	*EVA1C*	intron	no
1	3071769	*SCAF4*	intron	no
1	3531803	*TIAM1*	intron	no
1	3532855	*TIAM1*	intron	yes
1	3535643	*TIAM1*	intron	no
1	3537914	*TIAM1*	intron	yes
1	3540151	*TIAM1*	intron	yes
1	3860947	---		no
1	4002969	---		yes
1	4635165	---		yes
1	4833894	---		yes
1	4842016	---		yes
1	4859968	---		yes
1	4865611	---		yes
1	4945082	---		yes
1	5001661	---		yes
1	5020775	---		yes
1	5448996	*LOC100847531*	intron	no
1	5756595	*LOC100847531*	intron	no
1	5773698	*LOC100847531*	intron	no
1	5850349	*LOC100847531*	intron	no
1	6709101	---		yes
1	6712265	---		yes
1	6754744	---		no
