# Supplementary material for: Homozygous haplotype deficiency reveals deleterious mutations compromising reproductive and rearing success in cattle
Source: BMC Genomics. 2015 Apr 18;16(1):312. doi: 10.1186/s12864-015-1483-7 (PMC4403906; doi:10.1186/s12864-015-1483-7)
Supplement: Additional file 10: Table S5. — Primer sequences. [file 12864_2015_1483_MOESM10_ESM.pdf]

Sanger sequencing

NCBI SNP ID	Forward primer sequence (5'>3')	Reverse primer sequence (5'>3')	
rs379675307	ATGAGAAACGGTGCA CATGA	GCAGCATCAATGCCAC TAGA	
rs384285149	TATTCCGGGTGGGTGT TCTA	AGCCGATCACCCAGTA ATTG	
rs110793536	TCTCCACTCCCACTAC TAGGT	CTGAGCAGCAAGGAG ACATG	

TaqMan Genotyping

NCBI SNP ID	Primer sequence (5'>3')	Probe sequence (5'>3')	
rs110793536	F:TCCATTCCATATCAT CAGGAGGATTGA R:GAGTCTGGTGGTAC AGTTTTGAGT	VIC:CTACATCAGACCA GTTGGT FAM:CATCAGACCGGT TGGT	

KASP Genotyping

NCBI SNP ID	FAM primer sequence (5'>3')	HEX primer sequence (5'>3')	Reverse primer sequence (5'>3')
rs379675307	GTGATATCATCACTTC CTCTGAGTCT	GTGATATCATCACTTC CTCTGAGTGA	TGACCTGAGTTGTGTC AACATGATCATTT
rs384285149	CAGGGCGGAACACTG CATAGG	CAGGGCGGAACACTG CATAGA	GATGCTGGGGCTTCCA GCACTT
